# The MtrCDE Efflux Pump Contributes to Survival of *Neisseria gonorrhoeae* From Human Neutrophils and Their Antimicrobial Components

**DOI:** 10.3389/fmicb.2018.02688

**Published:** 2018-11-20

**Authors:** Jonathan W. Handing, Stephanie A. Ragland, Urmila V. Bharathan, Alison K. Criss

**Affiliations:** Department of Microbiology, Immunology, and Cancer Biology, University of Virginia, Charlottesville, VA, United States

**Keywords:** *Neisseria gonorrhoeae*, neutrophil, antimicrobial peptide, efflux pump, granule, neutrophil extracellular trap

## Abstract

The mucosal inflammatory response to *Neisseria gonorrhoeae* (Gc) is characterized by recruitment of neutrophils to the site of infection. Gc survives exposure to neutrophils by limiting the ability of neutrophils to make antimicrobial products and by expressing factors that defend against these products. The multiple transferable resistance (Mtr) system is a tripartite efflux pump, comprised of the inner membrane MtrD, the periplasmic attachment protein MtrC, and the outer membrane channel MtrE. Gc MtrCDE exports a diverse array of substrates, including certain detergents, dyes, antibiotics, and host-derived antimicrobial peptides. Here we report that MtrCDE contributes to the survival of Gc after exposure to adherent, chemokine-treated primary human neutrophils, specifically in the extracellular milieu. MtrCDE enhanced survival of Gc in neutrophil extracellular traps and in the supernatant from neutrophils that had undergone degranulation (granule exocytosis), a process that releases antimicrobial proteins into the extracellular milieu. The extent of degranulation was unaltered in neutrophils exposed to parental or *mtr* mutant Gc. MtrCDE expression contributed to Gc defense against some neutrophil-derived antimicrobial peptides but not others. These findings demonstrate that the Mtr system contributes to Gc survival after neutrophil challenge, a key feature of the host immune response to acute gonorrhea.

## Introduction

*Neisseria gonorrhoeae* (Gc) is the cause of the bacterial sexually transmitted infection gonorrhea. Gc is notorious for its prevalence (annual estimates in the United States and worldwide of 820,000 and 78 million cases, respectively), its resistance to numerous antibiotics, its association with infertility and other negative sequelae, and its ability to evade and subvert protective immune responses (Satterwhite et al., 2013; Unemo et al., 2016; Rice et al., 2017; Wi et al., 2017). Gc colonizes the human mucosal epithelium of the urogenital tract, pharynx, rectum, and conjunctiva. Epithelial infection stimulates the release of proinflammatory factors that recruit and activate neutrophils (Stevens and Criss, 2018; Stevens et al., 2018). Neutrophils are terminally differentiated, phagocytic granulocytes that make and release cationic antimicrobial peptides and antimicrobial proteins, as well as reactive oxygen species. These antimicrobial products are directed into nascent phagosomes to kill internalized microbes, or released into the extracellular milieu via granule exocytosis (degranulation) or in DNA-based neutrophil extracellular traps (NETs) to combat extracellular microbes (Nauseef and Borregaard, 2014). Although Gc induces neutrophil degranulation and NET release and is phagocytosed by neutrophils, these activities are not sufficient to clear Gc, and a subset of bacteria survive. Gc resists killing by neutrophils by limiting the release of antimicrobial products by neutrophils and by expressing proteins that defend against those products that the bacteria encounter (Palmer and Criss, 2018). Expression of enzymes that modify lipooligosaccharide, peptidoglycan turnover machinery, a NET-degrading nuclease, and zinc transporters have all been implicated in resistance of Gc to killing by neutrophils (Palmer and Criss, 2018).

The gonococcal multiple transferable resistance (Mtr) efflux pump exports a wide variety of structurally diverse antimicrobial agents, including cationic antimicrobial peptides, antibiotics, fatty acids, non-ionic detergents, and bile salts (Hagman et al., 1995, 1997; Shafer et al., 1998). MtrCDE is a member of the hydrophobic and amphiphilic efflux resistance-nodulation-division family of efflux pumps, which includes *Escherichia coli* AcrAB-TolC and *Pseudomonas aeruginosa* MexAB-OprM (Du et al., 2018). It is composed of inner membrane (MtrD) and outer membrane channels (MtrE), which are connected through a periplasmic membrane fusion lipoprotein (MtrC) (Hagman et al., 1995, 1997;Delahay et al., 1997). Efflux is dependent on energy supplied by the proton motive force that is transduced by MtrD (Janganan et al., 2013; Bolla et al., 2014). In addition to MtrC and MtrD, the MtrE outer membrane channel couples to other efflux pumps in Gc, including FarAB (fatty acid efflux) and MacAB (macrolide efflux), and cooperates with the MtrF inner membrane transporter (sulfonamide efflux) (Lee and Shafer, 1999; Veal and Shafer, 2003; Rouquette-Loughlin et al., 2005). *mtrC, mtrD*, and *mtrE* are found in an operon with a single promoter (Hagman et al., 1995). Expression of *mtrCDE* is directly regulated by a TetR family repressor, MtrR, and an AraC family activator, MtrA (Pan and Spratt, 1994; Hagman and Shafer, 1995; Rouquette et al., 1999). Clinically relevant mutations causing overexpression of MtrCDE can occur in the pump repressor (*mtrR*) or in the promoter region of the *mtrCDE* operon (Zarantonelli et al., 1999; Warner et al., 2008; Ohneck et al., 2011). These mutations confer increased resistance of Gc to antibiotics including penicillin, erythromycin, rifampin, and azithromycin (Hagman et al., 1995; Zarantonelli et al., 1999; Warner et al., 2008; Ohneck et al., 2011). Mutation of *mtrR* is a prerequisite for Gc to acquire porin IB variants (*penB*) that confer high-level resistance to penicillins and cephalosporins (Sparling et al., 1975; Veal et al., 2002; Olesky et al., 2006).

Generally, acquisition of antibiotic resistance alleles is associated with a fitness cost in bacteria. However, clinical and experimental evidence suggests the opposite is true for MtrCDE. Jerse et al. (2003) found that mutations in *mtrCDE* reduced Gc survival in the female murine genital tract, while mutations causing derepression of the *mtrCDE* operon that increase MtrCDE expression enhanced Gc survival (Warner et al., 2007, 2008). MtrCDE may contribute to Gc survival *in vivo* by protecting Gc from the antimicrobial effects of fatty acids and cationic antimicrobial peptides, such as cathelicidins (human LL-37 and mouse CRAMP-38) and other antimicrobials found at inflamed mucosal surfaces (Shafer et al., 1998; Warner et al., 2008). In agreement with this possibility, *mtrCDE* is expressed by Gc in the human urogenital tract of both men and women (McClure et al., 2015; Nudel et al., 2018).

Although MtrCDE is important for Gc infectivity *in vivo* and MtrCDE can efflux a variety of antimicrobials, including host-derived ones, the cellular contexts in which the MtrCDE system contributes to Gc pathogenesis are poorly understood. In this study, we tested the hypothesis that the MtrCDE efflux pump helps defend Gc from killing by neutrophils and their cationic antimicrobial proteins and peptides. In support of this hypothesis, expression of MtrCDE increased Gc viability after exposure to adherent, chemokine-treated primary human neutrophils. MtrCDE specifically enhanced extracellular survival of Gc in NETs, as well as associated with the neutrophil surface. The presence of MtrCDE variably affected resistance of Gc to antimicrobial peptides and proteins made by neutrophils. These findings reveal new roles for MtrCDE during Gc infection.

## Materials and Methods

### Bacterial Strains and Growth Conditions

Piliated, opacity protein (Opa)-deficient Gc of strain FA1090 served as the parent for this study (Ball and Criss, 2013). Gc was maintained on gonococcal medium base (BD Difco) with Kellogg’s supplements I + II (GCB) at 37°C, 5% CO_2_ (Kellogg et al., 1963). For neutrophil and antimicrobial protein survival experiments, Gc was grown and diluted in rich liquid medium (GCBL) containing Kellogg’s supplements and 0.042% sodium bicarbonate for multiple rounds of culture in order to enrich for predominantly mid-logarithmic phase bacteria (Criss et al., 2009).

### Construction of *mtr* Mutant and Complement Strains

To generate the *mtrE* mutant, the genomic region surrounding the *mtrE::kan* mutation (RD1) in strain FA19 (from Dr. William Shafer, Emory University) was amplified by PCR using the primer pair MTREF (5′-CGAAGACCAAGGCTTCGTTATGG-3′) and MTRER (5′-AATATTCAATGCCGACCGGACC-3′). The amplicon was introduced by natural transformation into FA1090 parent Gc, and transformants were selected on GCB containing 40 μg/ml kanamycin. Due to issues with PCR amplicon-mediated transformation at the time of strain construction, the *mtrC* and *mtrD* mutants in FA1090 parent Gc were generated by transformation and backcross with genomic DNA from FA19 *mtrC::kan* (KH12) or FA19 *mtrD::kan* (KH14) (from Dr. William Shafer, Emory University). Genomic DNA was introduced into FA1090 parent Gc by natural transformation, and transformants were selected on GCB containing 40 μg/ml kanamycin. Genomic DNA from one kanamycin-resistant transformant was isolated, verified to have a disrupted *mtr* allele by PCR (see below), and retransformed into the FA1090 parent Gc strain. This procedure was repeated one additional time, resulting in three consecutive backcrosses in total. Successful replacement of each of the wild-type Mtr genes with its mutated allele was confirmed by DNA sequencing of PCR amplicons that were generated using the following primer sets (located upstream of the *kan* insertion site for each allele): *mtrC*, MTRCF (5′-AGCCTTATCAGGAATGACTGG-3′) and MTRCR (5′-CCATAACGAAGCCTTGGTCTTCG-3′); *mtrD*, MTRDF (5′-CATTGGCAGTGTCGTCTTGC-3′) and MTRDR (5′-CTGCTGCAACAGAGGTCAAGG-3′). Sequencing primers were as follows: *mtrC*, MTRCSEQF (5′-TGCAACCCGTTCGAACATTCG-3′); *mtrD*, MTRDSEQF (5′-AACGGCGTGGAAGGTTTGG-3′); and *mtrE*, MTRESEQF (5′-TTGACCTCTGTTGCAGCAGC-3′). The FA1090 *mtrC* and *mtrD* mutants retained the 1-81-S2 *pilE* sequence, as shown by DNA sequencing of the *pilE* gene that was amplified by PCR using the primer pair PILRBS (5′-GGCTTTCCCCTTTCAATTAGGAG-3′) and SP3A (5′-CCGGAACGGACGACCCCG-3′), with PILRBS serving as sequencing primer. We verified that backcrossing did not result in Opa expression in the FA1090 *mtrC* and *mtrD* mutants, as determined by immunoblotting of bacterial lysates with the 4B12 pan-Opa monoclonal antibody.

**FIGURE 1:**
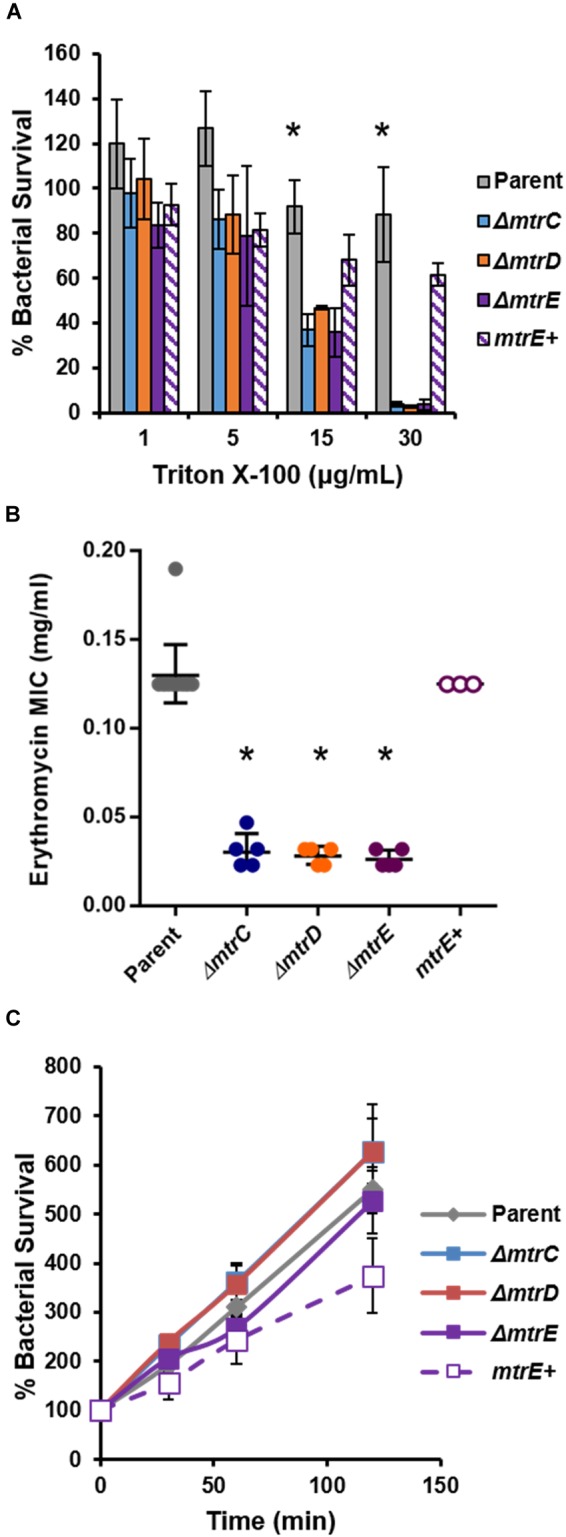
Characterization of MtrCDE efflux pump mutants in piliated, opacity protein-deficient Gc of strain FA1090. **(A)** “Opaless” parent, *ΔmtrC, ΔmtrD, ΔmtrE*, and *mtrE* complement (*mtrE^+^*) Gc were exposed to increasing concentrations of Triton X-100 or vehicle control for 1 h. Bacterial survival was calculated as the CFU enumerated after 1 h divided by CFU at the time of inoculation, expressed relative to survival in the vehicle control (100%). Data are presented as the mean ± SEM for 3–4 biological replicates. ^∗^*P* ≤ 0.05 for parent *vs.* each *mtr* mutant at the indicated concentration (one-way ANOVA followed by Tukey’s multiple comparisons test). **(B)** Minimal inhibitory concentrations (MIC) for erythromycin were calculated for parent, *ΔmtrC, ΔmtrD, ΔmtrE*, and *mtrE^+^* Gc. Data are presented as the geometric mean MIC ± SD for 3–11 biological replicates. ^∗^*P* < 0.0001 for parent vs. each *mtr* mutant and for *ΔmtrE* vs. *mtrE^+^* by one-way ANOVA followed by Tukey’s *post hoc* test. **(C)** Parent, *ΔmtrC, ΔmtrD, ΔmtrE*, and *mtrE^+^* Gc were inoculated into RPMI containing 10% FBS. Bacterial growth was calculated as the CFU enumerated at each time point divided by CFU at the time of inoculation (0 min). Data are presented as the mean ± SEM for 5–11 biological replicates.

To complement the FA1090 *mtrE::kan* mutant, Gc was transformed with the pKH35 complementation plasmid (Hamilton et al., 2005) (from Dr. Joseph Dillard, University of Wisconsin, Madison) containing an IPTG-inducible *mtrE* gene. The inducible allele was used in order to be able to titrate MtrE expression. *mtrE* was amplified from the genomic DNA of FA1090 parent Gc using the primer pair: MTRE_KpnI_COMPF (5′-TGC AGG TAC CGC AAA ATA CCG TCT GAG AAC C-3′) and MTRE_SpeI_COMPR (5′-CAG GAC TAG TCG GTT ATT TGC CGG TTT GG-3′). pKH35 and the PCR amplicon were digested with restriction enzymes KpnI and SpeI (New England Biolabs) and ligated together using T4 DNA ligase (New England Biolabs). Transformants were selected on 0.5 μg/ml chloramphenicol. Successful transformants were confirmed by DNA sequencing of a PCR amplicon corresponding to the intergenic *lctP-aspC* site in the Gc chromosome, using the primer pair aspC1 (5′-GCC GGA TGC GTC TTT GTA C-3′) and lctP (5′-GCG CGA TCG GTG CGT TCT-3′). MtrE expression was induced in the complement by adding 250 μg/ml IPTG into GCBL rich liquid medium for 2.5 h before experimental use. At this IPTG concentration, the *mtrE* complement showed statistically indistinguishable sensitivity to the FA1090 parent when challenged with the known MtrCDE efflux pump substrates Triton X-100 (Figure 1A) and LL-37 (Figure 5A). The *mtrE* complement is designated as *mtrE+.*

### Neutrophil Isolation

Neutrophils were purified from the heparinized venous blood of healthy human subjects using dextran sedimentation followed by separation on a Ficoll gradient and hypotonic lysis of erythrocytes, as previously described (Stohl et al., 2005). Neutrophils were resuspended in Dulbecco’s phosphate buffered saline (PBS; without calcium and magnesium; Thermo Scientific) containing 0.1% dextrose. Neutrophils were stored on ice for no longer than 2 h before use. All human subjects gave written informed consent in accordance with a protocol approved by the University of Virginia Institutional Review Board for Health Sciences Research and the Helsinki Declarations (IRB-HSR protocol #13909).

### Bacterial Growth in Rich Liquid Media

Parent, *mtrC* mutant, *mtrD* mutant, *mtrE* mutant, and *mtrE+* complement Gc were grown in GCBL with NaHCO_3_ as described above. Mid-logarithmic phase Gc was inoculated into RPMI (Mediatech) with 10% fetal bovine serum (FBS, heat-inactivated; Thermo Scientific) at 1 × 10^6^ colony-forming units (CFU)/ml final concentration in replicate wells of a 24-well plate. Gc was incubated at 37°C, 5% CO_2_. At the start of the experiment (time = 0 min) and indicated times thereafter, well contents were mixed thoroughly, and an aliquot from each well was serially diluted and plated on GCB agar. Bacterial growth at each time point is reported relative to CFU enumerated at 0 min for each strain, which is set to 100%.

### Bacterial Survival From Human Neutrophils

Neutrophils (10^6^ cells per coverslip) were resuspended in RPMI containing 10% FBS and 10 nM interleukin-8 (IL-8, carrier free; R&D Systems). Neutrophils were added to tissue culture-treated plastic coverslips (Sarstedt) in 24-well plates and allowed to adhere for 1 h at 37°C in 5% CO_2_. Neutrophils were then challenged with parent, *mtrC* mutant, *mtrD* mutant, *mtrE* mutant, or *mtrE+* complemented mid-logarithmic phase Gc at a multiplicity of infection of 1 as described previously (Criss et al., 2009). At indicated time points, neutrophils were lysed in 1% saponin, and lysates were diluted and plated on GCB agar. CFU were enumerated from lysates after 20–24 h growth, and percent survival was calculated relative to the CFU enumerated at the start of the experiment (time = 0 min, set to 100%).

### Intracellular and Extracellular Bacterial Viability After Neutrophil Challenge

Baclight viability dyes (Invitrogen) were used in conjunction with soybean lectin-Alexa Fluor 647 conjugate (Life Technologies) to discriminate intracellular and extracellular Gc in association with neutrophils after 1 hr of infection as described previously (Johnson and Criss, 2013a).

### Bacterial Survival From NETs

Neutrophils (10^6^ cells per coverslip) in phenol red-free RPMI (Mediatech) with 5% FBS were treated with phorbol myristate acetate (PMA, 10 nM; Sigma), and allowed to adhere to tissue culture-treated plastic coverslips for 30 min at 37°C, 5% CO_2_. Neutrophils were treated with 10 μg/ml cytochalasin D (Sigma) in the presence or absence of 1 U/ml DNase I (New England Biolabs) for 20 min at 37°C, 5% CO_2_. Neutrophils were exposed to mid-logarithmic phase Gc at an MOI = 1 and incubated for 1 h at 37°C, 5% CO_2_. Percent survival was calculated as the CFU enumerated after 1 hr of neutrophil exposure divided by the CFU added at 0 min.

### Neutrophil Degranulation by Flow Cytometry

Surface presentation of granule-specific markers was measured by flow cytometry essentially as described in Ragland et al. (2017). Briefly, adherent neutrophils exposed to Gc were lifted with 5 mM EDTA, washed with DPBS containing 0.1% dextrose, and simultaneously stained with PE-CD63 (Biolegend) and APC-CD66b (Biolegend) as indicators of primary and secondary granule exocytosis, respectively, or respective isotype controls (Biolegend PE-IgG1, κ and Biolegend APC-IgM, κ). Data were acquired using a FACSCalibur Benchtop Analyzer and analyzed using FlowJo software. The geometric means of fluorescence intensity for PE and APC were calculated from a gate that includes all granulocytes by side scatter and forward scatter.

### Bacterial Survival After Exposure to Antimicrobial Components

Mid-logarithmic phase Gc was incubated with the antimicrobial component as described below at 37°C, 5% CO_2_. CFU were enumerated at 0 min and after incubation for the indicated time. Percent survival was calculated as CFU_afterincubation_ ÷ CFU_0 min_ for each concentration of antimicrobial component and expressed relative to Gc survival in the vehicle control, which was standardized to 100%.

#### Triton X-100

Triton X-100 (Sigma) was diluted in ddH_2_O. Gc was incubated in Triton X-100 in 0.5x GCBL for 1 h.

#### Supernatant From Degranulated Neutrophils

Adherent neutrophils (10^6^ cells per coverslip) were treated with PMA (10 nM; Sigma) in RPMI for 30 min at 37°C in 5% CO_2_. The supernatant was filtered (0.2 μm) to remove neutrophils. Gc (10^6^ CFU) was incubated in 0.2 ml supernatant or RPMI media control for 1 h.

#### LL-37 Cathelicidin

LL-37 was provided by Dr. William Shafer, Emory University and was diluted in ddH_2_O. Gc was incubated in LL-37 in 0.2x GCBL for 1 h.

#### Bactericidal Permeability-Increasing Protein

BPI (Novateinbio) was diluted in ddH_2_O. Gc was incubated with BPI in 0.5x GCBL for 2 h.

#### Lysozyme

Human lysozyme (Sigma) was diluted in ddH_2_O. Gc was incubated with lysozyme in 0.5x GCBL for 3 h.

#### Azurocidin

Azurocidin (Sigma) was diluted in ddH_2_O. Gc was incubated in azurocidin in 0.5x GCBL for 45 min.

### Antibiotic Minimal Inhibitory Concentration

Gc susceptibility to erythromycin and vancomycin were measured using *E*-test strips (Biomérieux) as in Ragland et al. (2017).

### Statistics

Values are the mean ± the standard error of at least three, independent replicates performed on different days, except for MIC, which was measured as the geometric mean. Significance was assessed using Student’s *t-*test, or for multiple comparisons, one-way ANOVA followed by Tukey’s *post hoc* test. A *P*-value of < 0.05 was considered significant.

## Results

### Contribution of the MtrCDE Efflux Pump to Gc Survival From Adherent Human Neutrophils

To test the role of the MtrCDE system in Gc defense against human neutrophils and neutrophil-derived antimicrobials, we introduced insertional mutations in *mtrC, mtrD*, and *mtrE* into the “Opaless” constitutively piliated, Opa protein-deficient Gc of strain FA1090 (Ball and Criss, 2013). Opaless Gc was used as the parent for this study because piliated, Opa protein-deficient Gc exhibits enhanced survival in the presence of human neutrophils, and both pili and Opa protein expression vary at high frequency (Criss et al., 2009; Ball and Criss, 2013; Stohl et al., 2013; Rotman and Seifert, 2014). The *mtrE* mutant was complemented with the *mtrE* gene under the control of an isopropyl-β-D-galactosidase (IPTG)-inducible promoter (*mtrE^+^*), and was introduced into an ectopic locus in the Gc chromosome. We verified the MtrCDE system was functionally inactivated in the *mtrC, mtrD*, and *mtrE* mutants by their increased sensitivity to killing by two MtrCDE efflux pump substrates, Triton X-100 and erythromycin (Figures 1A,B). Complementation rescued the susceptibility of the *mtrE* mutant to the two substrates (Figures 1A,B). Inactivation of MtrC, D, or E in the Opaless background had no effect on bacterial growth in rich liquid medium (Figure 1C).

**FIGURE 2:**
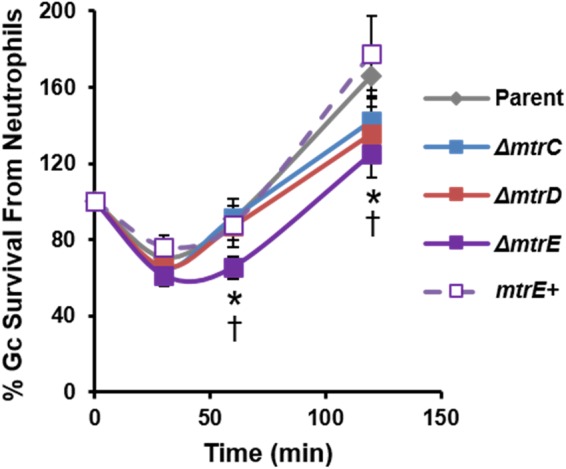
Contribution of the MtrCDE efflux pump to Gc survival from adherent human neutrophils. Parent, *ΔmtrC, ΔmtrD, ΔmtrE*, and *mtrE* complement (*mtrE+*) Gc were exposed to adherent, IL-8-treated primary human neutrophils for the indicated times. Gc survival was calculated by CFU enumeration at each time point and expressed as the percent of CFU enumerated at 0 min. Data are presented as the mean ± SEM for 5–11 biological replicates. ^∗^*P* ≤ 0.05 for *ΔmtrE* vs. parent Gc, and ^†^*P* ≤ 0.05 for *ΔmtrE* vs. *mtrE* complement Gc (Student’s two-tailed *t*-test).

We evaluated the contribution of MtrCDE to Gc survival from neutrophils, using adherent, interleukin-8 treated primary human neutrophils to approximate the tissue-localized state of neutrophils after migration to sites of infection (Stevens and Criss, 2018). Parent, *mtr* mutant, and *mtrE* complement Gc were incubated with neutrophils, and bacterial CFU from neutrophil lysates were enumerated over time (Figure 2). There was a small but statistically significant decrease in recovery of *mtrE* mutant Gc exposed to neutrophils for 60 and 120 min compared with the parent (Figure 2, solid purple line). Recovery of the *mtrE* mutant was restored by complementation (Figure 2, dotted purple line). In contrast, the *mtrC* and *mtrD* mutants were not significantly different from parent Gc in their recovery from adherent human neutrophils over time (Figure 2, blue and red lines).

### The MtrCDE Efflux Pump Enhances Gc Survival From Killing by Neutrophils in the Extracellular Space

**FIGURE 3:**
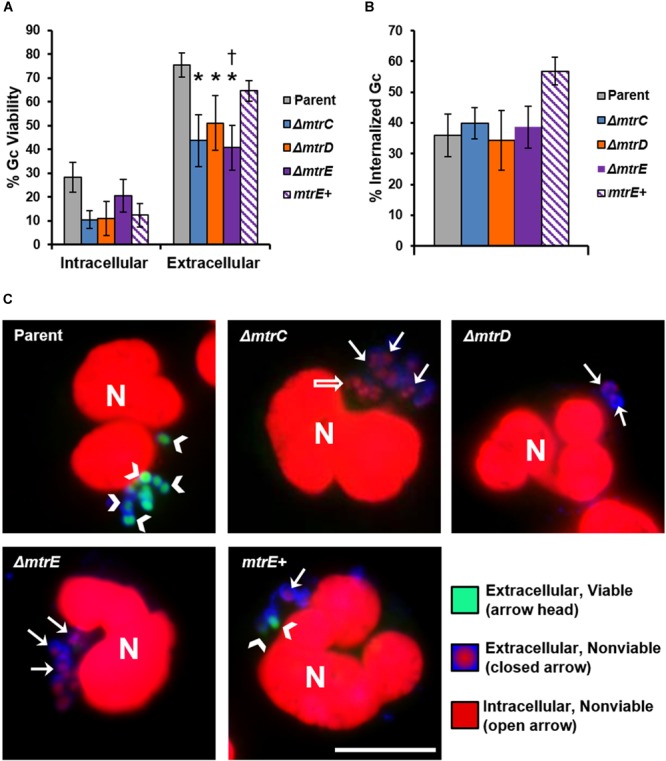
MtrCDE enhances extracellular Gc survival in the presence of adherent human neutrophils. Parent, *ΔmtrC, ΔmtrD, ΔmtrE*, and *mtrE* complement (*mtrE+*) Gc were exposed to adherent, IL-8-primed primary human neutrophils for 1 h. Extracellular Gc were identified with Alexa Fluor 647-conjugated soy bean lectin (blue), and viable and nonviable Gc were identified with SYTO9 (green) and propidium iodide (red), respectively. **(A)** Percent viable Gc was determined by dividing the number of viable Gc by the total Gc in the intracellular or extracellular compartment for 100–150 neutrophils. Data are presented as the mean ± SEM for 5 biological replicates. ^∗^*P* ≤ 0.05 for each *mtr* mutant vs. parent, and ^†^*P* ≤ 0.05 for *ΔmtrE* vs. *mtrE+* complement Gc in the extracellular milieu (paired, two-tailed Student’s *t*-test). **(B)** The percent of cell-associated Gc that was internalized was calculated by dividing the total number of intracellular Gc by the total number of cell-associated Gc for 100–150 neutrophils. Data are presented as the mean ± SEM for 5 biological replicates. **(C)** Representative fluorescence micrographs per infection condition as indicated in the upper left corner of each image. Arrowheads indicate extracellular viable Gc, closed arrows indicate extracellular nonviable Gc, and open arrows indicate intracellular nonviable Gc. No intracellular viable Gc are apparent in these images. Neutrophil nuclei (N) are also propidium iodide-positive. Scale bar, 10 μm.

The CFU recovery assay used in Figure 2 reports on the survival of both intracellular and extracellular Gc. In order to directly assess how MtrCDE contributes to Gc survival in each of these locations, we used fluorescent viability dyes alongside a fluorescent lectin to detect extracellular bacteria. Bacteria with permeant membranes stain with the DNA dye propidium iodide, while bacteria with intact membranes exclude this dye and are instead counterstained with SYTO9 (Johnson and Criss, 2013a). In agreement with our previous findings, the viability of Gc was reduced inside neutrophils compared with extracellular, cell surface-associated bacteria (Criss et al., 2009; Johnson and Criss, 2013b). Survival of extracellular *mtrC, mtrD*, and *mtrE* mutant Gc was significantly decreased compared with parent bacteria, and survival of the *mtrE* mutant was rescued by complementation (Figure 3A, representative images in Figure 3C). In contrast, there were no statistically significant differences in survival of *mtr* mutant Gc inside human neutrophils compared to parent bacteria (Figure 3A). There were also no differences in the ability of neutrophils to bind or phagocytose parent, *mtr* mutant, or *mtrE* complement Gc (Figure 3B). These results indicate that the MtrCDE efflux pump is specifically important for defending Gc from extracellular killing by neutrophils.

**FIGURE 4:**
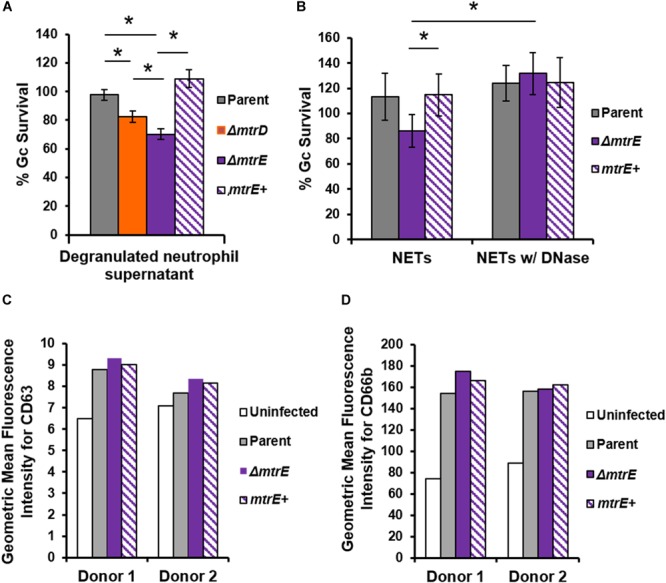
The MtrE outer membrane efflux pump channel contributes to extracellular Gc survival from degranulated neutrophils and from neutrophil extracellular traps. **(A)** Parent, *ΔmtrD, ΔmtrE*, and *mtrE* complement (*mtrE*+) Gc were exposed for 1 h to supernatants from PMA-stimulated human neutrophils. Percent Gc survival was calculated as in Figure 1A. Data are presented as the mean ± SEM for 3–9 biological replicates. ^∗^*P* ≤ 0.05 for indicated comparisons (Student’s two-tailed *t*-test). **(B)** Parent, *ΔmtrE*, and *mtrE* + Gc were exposed for 1 h to neutrophils treated with PMA and cytochalasin D to produce NETs, in the presence or absence of bovine DNase. Percent Gc survival was calculated by dividing CFU enumerated after 1 h by the inoculum at 0 h. Data are presented as the mean ± SEM for 3 biological replicates. ^∗^*P* ≤ 0.05 for indicated comparisons (Student’s two-tailed *t*-test). The *P-*value for the difference between parent and *ΔmtrE* Gc in the presence of NETs without DNase was 0.06. **(C,D)** Adherent, IL-8 treated human neutrophils were exposed to parent, *ΔmtrE*, or *mtrE*+ Gc for 1 h, or left uninfected in media. Surface exposure of the primary granule component CD63 **(C)** or the secondary granule component CD66b **(D)** was measured by flow cytometry and reported as the geometric mean fluorescence. Results from two independent blood donors are presented.

Neutrophils have two modes of extracellular killing: release of antimicrobial agents by granule exocytosis (degranulation) and via NETs. We thus measured survival of Gc after exposure to both extracellular environments. For these and subsequent assays, the *mtrC* mutant was not used since it was expected to phenocopy the *mtrD* mutant. Survival of *mtrD* and *mtrE* mutant Gc was significantly reduced after incubation with supernatant collected from neutrophils that were stimulated to degranulate by treatment with PMA (Figure 4A). Survival of the *mtrE* mutant was rescued by complementation (Figure 4A). When Gc was exposed to neutrophils that had released NETs after PMA and cytochalasin D treatment (Handing and Criss, 2015), the *mtrE* mutant was significantly reduced in survival compared with the parent and the *mtrE+* complement (Figure 4B). Survival of the *mtrE* mutant was also rescued by addition of exogenous DNase to degrade NETs (Figure 4B). There was no difference in the ability of parent or *mtrE* mutant Gc to stimulate neutrophil degranulation, as measured by flow cytometry for surface exposure of primary/azurophilic (CD63) or secondary/specific granule proteins (CD66b) (Figures 4C,D). We conclude that the Mtr efflux pump, especially the MtrE channel, enhances the resistance of Gc to extracellular mechanisms of killing used by neutrophils.

### The MtrCDE Efflux Pump Defends Gc From Select Antimicrobial Proteins and Peptides Made by Human Neutrophils

**FIGURE 5:**
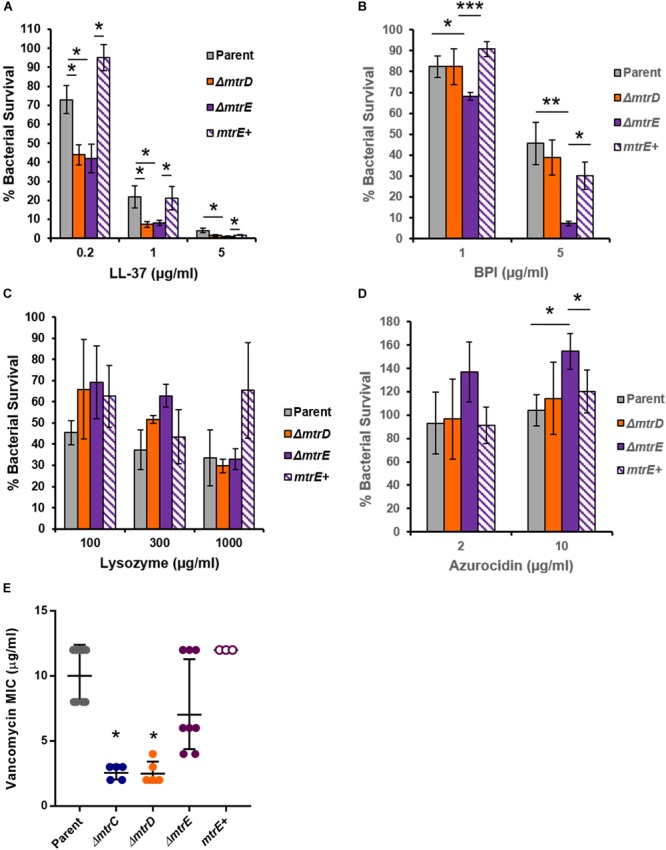
MtrCDE efflux pump components enhance Gc survival in the presence of selected neutrophil granule components. **(A)** Parent, *ΔmtrD, ΔmtrE*, and *mtrE* complement (*mtrE+*) Gc were exposed to LL-37 at the indicated final concentrations for 1 hr. Percent Gc survival was determined as in Figure 1A. Data are presented as the mean ± SEM for 3–9 biological replicates. ^∗^*P* ≤ 0.05 for the indicated comparisons (Student’s two-tailed *t-*test). **(B)** Parent, *ΔmtrD, ΔmtrE*, and *mtrE+* Gc were exposed to BPI at the indicated final concentrations for 45 min. Percent Gc survival was determined as in Figure 1A. Data are presented as the mean ± SEM for 3–7 biological replicates. ^∗^*P* ≤ 0.05, ^∗∗^*P* ≤ 0.025, and ^∗∗∗^*P* ≤ 0.01 for the indicated comparisons (Student’s two-tailed *t-*test). **(C)** Parent, *ΔmtrD, ΔmtrE*, and *mtrE+* Gc were exposed to lysozyme at the indicated final concentrations for 3 h. Percent Gc survival was determined as in Figure 1A. Data are presented as the mean ± SEM for 3–4 biological replicates. **(D)** Parent, *ΔmtrD, ΔmtrE*, and *mtrE+* Gc were exposed to azurocidin at the indicated final concentrations for 45 min. Percent Gc survival was determined as in Figure 1A. Data are presented as the mean ± SEM for 3–6 biological replicates. ^∗^*P* ≤ 0.05 for the indicated comparisons (Student’s two-tailed *t* test). **(E)** MICs for vancomycin were calculated for parent, *ΔmtrC, ΔmtrD, ΔmtrE*, and *mtrE^+^* Gc. Data are presented as the geometric mean MIC ± SD for 3–9 biological replicates. ^∗^*P* < 0.0001 for *ΔmtrC* vs. parent, *ΔmtrE*, or *mtrE^+^* and *ΔmtrD* vs. parent, *ΔmtrE*, or *mtrE^+^* by one-way ANOVA followed by Tukey’s *post hoc* test. Parent, *ΔmtrE*, and *mtrE^+^* Gc were not significantly different from one another.

Given that Mtr expression helped Gc resist killing by the degranulated supernatant of human neutrophils (Figure 4A), we sought to identify neutrophil products that were substrates for efflux by MtrCDE. Parent, *mtrD* mutant, *mtrE* mutant, and *mtrE* complement Gc were incubated with purified neutrophil antimicrobial proteins, and survival was measured by enumerating CFU after incubation (see Materials and Methods). In accordance with previous reports (Shafer et al., 1998), we found that the *mtrD* and *mtrE* mutant in the Opaless background were significantly more sensitive to killing by LL-37 than either parent or *mtrE+* complement Gc (Figure 5A). In contrast, the *mtrE* mutant, but not the *mtrD* mutant, was more sensitive to killing by bactericidal-permeability increasing protein (BPI) (Figure 5B). The *mtrD* and *mtrE* mutants showed no difference in survival compared to parent or *mtrE*+ Gc after incubation with human lysozyme (Figure 5C).

Unexpectedly, the *mtrE* mutant was more resistant to the primary granule antimicrobial protein azurocidin than the parent, *mtrD* mutant, or *mtrE+* complement (Figure 5D). This finding led us to explore if there were other antimicrobial compounds where the *mtrD* mutant was more susceptible than the *mtrE* mutant. Gram-negative bacteria like Gc are intrinsically resistant to vancomycin, unless their outer membrane is somehow breached (Ragland et al., 2017). The *mtrC* and *mtrD* mutants had a significantly lower MIC to vancomycin compared with parent or *mtrE* mutant Gc (Figure 5E). Unlike results with azurocidin, the parent, *mtrE* mutant, and *mtrE+* complement all had similar sensitivity to vancomycin (Figure 5E).

Together, these findings show that the MtrCDE efflux pump and the MtrE outer-membrane channel contribute in different ways to defense against neutrophil-derived antimicrobial proteins and peptides.

## Discussion

The gonococcal *mtr* system was first identified 45 years ago (Maness and Sparling, 1973). While many of the diverse substrates subject to efflux by MtrCDE have been identified, when and where Gc employs the MtrCDE system during infection have not been fully resolved. In this study, we showed that the MtrCDE efflux pump contributes to resistance of Gc to killing by human neutrophils. Specifically, MtrCDE enhances Gc survival extracellularly, in NETs and upon exposure to neutrophil products that are released by degranulation. Using purified cationic antimicrobial proteins and peptides made by neutrophils, we identified four phenotypes associated with MtrCDE in Gc: (1) MtrCDE-dependent sensitivity (e.g., LL-37), (2) MtrE-dependent but MtrD-independent sensitivity (e.g., BPI), (3) MtrD-dependent but MtrE-independent sensitivity (e.g., azurocidin, vancomycin), (4) no effect of MtrCDE (e.g., lysozyme). These findings suggest a location- and component-specific defense conferred by the MtrCDE efflux pump when Gc is confronted by neutrophils, as during acute human infection.

MtrCDE improves Gc survival in the murine vaginal tract (Jerse et al., 2003; Warner et al., 2007, 2008), suggesting that the efflux pump may be involved in export of antimicrobials released by host mucosal epithelial cells. We demonstrated that MtrCDE protects Gc from extracellular antimicrobials released from human neutrophils, and protects Gc from killing by NETs that contain various antimicrobial proteins. It is notable that the *mtrC* and *mtrD* mutants showed a survival defect when enumerated by microscopy for bacterial viability, while they were not statistically different from the parent by CFU enumeration. This is in contrast to the *mtrE* mutant, which showed a significant decrease in survival by both assays. The CFU assay accounts for the viability of all bacteria in the infection: intracellular, extracellularly cell-associated, and free in the extracellular milieu. In contrast, free extracellular bacteria are lost when the infected neutrophils are processed for fluorescence microscopy with the viability dyes. The *mtrD* mutant was significantly less susceptible than the *mtrE* mutant to killing by a mix of antimicrobial peptides and proteins that were released from degranulated neutrophils (Figure 4A). Thus we anticipate that the decreased viability of only the *mtrE* mutant by the CFU assay is due to this non-cell-associated, extracellular fraction of bacteria.

Although neutrophil phagosomes that have fused with granules contain high concentrations of antimicrobial peptides and proteins, MtrCDE was dispensable for survival of Gc inside neutrophils. One explanation for this surprising observation is that Gc-containing phagosomes do not contain substrates for efflux through MtrCDE. We previously reported that Opa-negative Gc, as used in this study, resides in an immature phagosome that has undergone fusion with secondary but not primary granules (Johnson and Criss, 2013b). Secondary granules contain hCAP18, which is processed by primary granule proteases to generate the mature antimicrobial peptide LL-37 (Sorensen et al., 2001). Thus a primary granule-negative phagosome is not expected to contain mature LL-37. BPI and azurocidin are also predominantly found in primary granules (Nauseef and Borregaard, 2014). However, we did not measure any differences in MtrCDE-dependent Gc survival inside neutrophils when the bacteria were first opsonized with IgG, which directs them into primary granule-positive phagolysosomes (Johnson and Criss, 2013b) (data not shown). Alternatively, antimicrobial proteins could reach a concentration inside the Gc-containing phagosome that overwhelms MtrCDE efflux activity, such that no susceptibility phenotype is observed for *mtr* mutants. However, we do not favor this possibility because we have not measured significant differences in the intracellular viability profile of wild-type Gc of the FA1090 and MS11 backgrounds; FA1090 naturally expresses lower MtrCDE levels compared to MS11 due to natural mutations in *mtrA* and *mtrR*, respectively (Rouquette et al., 1999; Ohneck et al., 2011). Studies to test the role of MtrCDE in strains that more robustly express the efflux pump, to measure *mtr* expression in Gc residing inside neutrophils, and to identify the cohort of antimicrobials inside Gc phagosomes would help to clarify this issue.

NET formation is an important component of the host innate immune response to many bacterial pathogens (Brinkmann et al., 2004). Both Gc and *Neisseria meningitidis* can induce NET release from human neutrophils *in vitro* (Lappann et al., 2013; Gunderson and Seifert, 2015; Juneau et al., 2015). Gc and *N. meningitidis* modify the lipid A portion of their lipooligosaccharide (LOS) with phosphoethanolamine (PEA), which reduces susceptibility to killing by several neutrophil-derived antimicrobial components including LL-37, and protects them from killing within NETs (Lappann et al., 2013; Handing and Criss, 2015; Juneau et al., 2015; Kahler et al., 2018). We have shown here that the MtrCDE efflux pump also contributes to Gc survival in the presence of neutrophils induced to make NETs. In contrast, MtrCDE does not enhance survival of *N. meningitidis* exposed to NETs, which may be due to virulence factors such as the capsule that additionally defend against NETs (Lappann et al., 2013).

Many of the antimicrobial peptides and proteins made by neutrophils and released by degranulation have amphipathic characteristics, which are a feature shared by the structurally diverse substrates of the MtrCDE efflux pump. Amphipathic molecules possess regions of hydrophobic and hydrophilic elements that tend to favor interaction with biological membranes (Wiesner and Vilcinskas, 2010). The LL-37 antimicrobial peptide, which is made by neutrophils as well as epithelial cells, is an amphipathic molecule and a well-described MtrCDE efflux pump substrate. Many other neutrophil antimicrobial proteins and peptides have amphipathic characteristics, including α-defensins, lysozyme, BPI, CAP37/azurocidin, lactoferrin and cathepsin G, yet not all of these showed MtrCDE-dependent effects on Gc survival. Susceptibility to these antimicrobial peptides and proteins was not correlated with their molecular weight and potential ability to be exported through the 22 Å MtrE pore (Lei et al., 2014). For instance, the *mtrE* mutant did not have increased sensitivity to lysozyme (14.5 kDa), but was more sensitive to BPI (55 kDa). Given that the MtrE outer membrane channel couples to multiple efflux pump systems in Gc (Lee and Shafer, 1999; Veal and Shafer, 2003; Rouquette-Loughlin et al., 2005), these findings either suggest an MtrE-dependent but MtrCD-independent efflux pump exports the antimicrobial proteins, multiple MtrE-dependent efflux pumps work in concert for export, or the presence of MtrE changes the properties of the outer membrane to render it more resistant to antimicrobial protein and peptide attack. Future studies will explore how these and other efflux systems contribute to Gc susceptibility to neutrophil-derived antimicrobials.

One surprising finding arising from this study was that loss of the MtrE outer membrane channel enhanced, not reduced, Gc survival after exposure to azurocidin. Along these lines, loss of MtrE had no effect on sensitivity to vancomycin, but loss of MtrC and MtrD did. In both cases the phenotype of the *mtrE* mutant was rescued by complementation, suggesting the effects were due to MtrE and not a second site mutation. This raises the intriguing possibility that MtrE is exploited by selected antimicrobials as a portal across the outer membrane. This is counterintuitive since efflux pumps should work unidirectionally in export, yet small molecules like antibiotics and heme can transit across the outer membrane through channels such as the PorB porin and the PilQ pilus channel (Chen et al., 2004; Zhao et al., 2005; Olesky et al., 2006; Lindberg et al., 2007). Blocking antibodies, channel inhibitors, or mutations that sterically hinder channel activity are all approaches that can be used to test these possibilities (Janganan et al., 2011, 2013; Wang et al., 2018). It is also possible that loss of *mtrE* has secondary effects on protein or lipooligosaccharide abundance or composition of the outer membrane, to render Gc more resistant to a subset of antimicrobials.

The *mtrCDE-mtrR* locus has been recently described as a hotspot for genetic recombination (Wadsworth et al., 2018), and antibiotic-resistant strains of Gc frequently carry mutations that inactivate MtrR, leading to increased MtrCDE expression (Rice et al., 2017). These variations may translate into overall effects on Gc fitness *in vivo*, as seen in the female murine genital tract and potentially in the human urethral Gc challenge model (Jerse et al., 2003; Warner et al., 2007, 2008; Hobbs et al., 2011). Based on our findings, the antimicrobial activities of neutrophils are yet another stressor with which Gc contends by using the MtrCDE efflux pump. Interestingly, Nudel et al. (2018) recently reported that *mtrCDE* expression was twofold higher in Gc isolated from the male urethra compared with the female genital tract, including in individuals carrying the same bacterial strain. Therefore, we posit that changes in expression of MtrCDE assist Gc in successfully colonizing host sites that differ in neutrophil load and neutrophil activation state, including male vs. female hosts, lower vs. upper genital infection, and genital vs. extragenital sites of infection.

## Author Contributions

JH, SR, and AC contributed to the conception and design of the study. JH, SR, and UB performed the experiments and analyzed the results. JH, SR, and AC wrote the manuscript. All authors performed statistical analyses and contributed to the editing of the manuscript prior to submission and read and approved the submitted version of the manuscript.

## Conflict of Interest Statement

The authors declare that the research was conducted in the absence of any commercial or financial relationships that could be construed as a potential conflict of interest.
